# Morphological and molecular characterization of *Hoplolaimus pararobustus* (Schuurmans Stekhoven and Teunissen, 1938) Sher 1963 with its first report on *Zea mays* roots in Namibia

**DOI:** 10.21307/jofnem-2020-124

**Published:** 2021-01-13

**Authors:** Mariette Marais, Esther van den Berg, Hendrika Fourie, Milad Rashidifard

**Affiliations:** 1 National Collection of Nematodes, Biosystematics, ARC-Plant Heath and Protection, Private Bag X134, Queenswood, 0121, South Africa; 2 Unit for Environmental Sciences and Management, North-West University, Private Bag X6001, Potchefstroom, 2520, South Africa

**Keywords:** *Hoplolaimus*, *H. pararobustus*, Lance nematode, Maize, Morphology, Molecular identification, Ribosomal DNA, Taxonomy, Phylogeny

## Abstract

In the summer of 2018, specimens of a *Hoplolaimus* population were extracted from a maize root sample collected near Stampriet, Namibia. This population was identified as *Hoplolaimus pararobustus* and is described and illustrated based on its morphological, morphometric, and molecular characteristics. To our knowledge, this is the first report *H*. *pararobustus* from maize roots. Females of the population had a mean body and stylet length of 1,100 µm and 36 µm, respectively. Esophagus with three nuclei in three pharyngeal glands. Lateral field reduced, ranging from a very faint line to just breaks in striae. The males were shorter than the females with a mean body length of 925 µm and the stylet slightly shorter, with a mean length of 34 µm. Phylogenetic analyses using partial sequences of 18 S and the expansion fragment D2–D3 of 28 S rDNA genes showed the close relation of this species and *H. columbus*. This Namibian population of *H. pararobustus* is the first *Hoplolaimus* species from Africa to be molecularly characterized.

Lance nematodes, *Hoplolaimus* spp., are robust nematodes with a very distinct lip region and well-developed stylet with distinctly shaped stylet knobs (Fortuner, 1991) that feed on a wide range of plants and have a global distribution. Bae et al. (2009) reported that Handoo and Golden (1992) recognized 29 species in the genus *Hoplolaimus* Von Daday, 1905. Siddiqi (2000) listed 32 species in three subgenera with two species *Hoplolaimus johani* Tiwari, Mishra and Malhotra, 2001 and *Hoplolaimus caudifurcatus* Tiwari, Mishra and Malhotra, 2001, described in 2001 (Tiwari et al., 2001). Since the 2009 publication, three species namely *Hoplolaimus bachlongviensis* Nguyen, Bui and Trinh, 2015, *Hoplolaimus puriensis* Ali, Shaheen and Pervez, 2009 and *Hoplolaimus smokyensis* Ma, Robbins, Bernard, Holgun and Agudela, 2019 were described from Vietnam, India, and the United States of America, respectively (Ali et al., 2009; Nguyen et al., 2015; Ma et al., 2019). *Hoplolaimus pararobustus* (Schuurmans Stekhoven and Teunissen, 1938) Sher (1963) was described from the Democratic Republic of the Congo and since then, the species had been reported from 25 African countries, including Namibia (Loubana et al., 2007; EPPO, 2020).

*Hoplolaimus pararobustus* is associated with many plant species like grasses from bowling greens and lawns, fynbos, *Adansonia digitata* L., *Ananas comosus* (L.) Merr., *Annona reticulata* L., *Amaranthus* sp., *Beta vulgaris* L., *Camellia sinensis* (L.) Kuntze, *Carica papaya* L., *Chloris gayana* Kunth, *Coffea arabica* L., *Citrus* sp., *Daucus carotae* L., *Digitaria abyssinica* (A.Rich.) Stapf, *Dioscorea* spp., *Elaeis* spp., *Eucalyptus* sp., *Gossypium hirsutum* L., *Mangifera indica* L., *Musa* spp., *Oryza sativa* L., *Paspalanum notatum* Flügge, Phaseolus vulgaris L., *Psidium guajava* L., *Pueraria phaseoloides* var. *javanica* (Benth.) Baker; *Triticum aestivum* L., *Saccharum officinarum* L., *Solanum tuberosum* L., *Vigna unguiculata* (L.) Walp., *Vitis vinifera* L., and *Zea mays* L. (Caveness, 1967; Van den Berg and Heyns, 1970; Siddiqi, 1974; Bridge et al., 1995; Vovlas and Lamberti, 1985; CABI, 2014; Sikora et al., 2018). This semi-endoparasitic nematode (Yeates et al., 1993) has a broad distribution in Namibia, according to the South African Plant-Parasitic Nematode Survey (SAPPNS) database (De Waele et al., 1998; Marais et al., 2017) and it had been reported in soil around *Acacia millefolia* S.Watson, *Brassica oleracea* L., *Cenchrus ciliaris* L., *Cucurbita maxima* Duchesne, *Gossypium hirsutum* L., *Medicago sativa* L., *Pennisetum glaucum* (L.) R.Br., *Solanum lycopersicum* L., *Vitis vinifera* L., and Z. *mays*.

*Hoplolaimus pararobustus* has been reported from inside roots of banana (Whitehead, 1959) giving rise to dark-brown pustules that eventually result in necrotic cortical tissue situated around the heads of the feeding sites of the nematodes and eventually elongated ulcerated lesions on the roots (Siddiqi, 1974). It has also been reported, at a population density of 200 individuals per gram of tissue, in corms and roots of banana where they were likely to cause damage to the crop (Sikora et al., 2018). Phylogenetic analysis of *Hoplolaimus* spp. using D2–D3 expansion of 28 S and internal transcribed spacer (ITS1) ribosomal DNA sequences resolved the phylogeny of the genus and were useful in molecular identification of *Hoplolaimus* spp. (Bae et al., 2008). In addition, the PCR-RFLP method was applied by different researchers to evaluate the genetic diversity of *Hoplolaimus* spp. (Robbins et al., 2009; Bae et al., 2009). Later, a species-specific primer was developed to distinguish *Hoplolaimus stephanus* Sher, 1963 from another similar species *viz. Hoplolaimus galeatus* (Cobb, 1913) Thorne, 1935 (Ma et al., 2011). Moreover, sequences of the actin gene were successfully used for phylogenetic studies of *Hoplolaimus* spp. (Ma et al., 2011). High genetic variability among the *Hoplolaimus* populations in soybean-growing areas in the USA was reported when their genetic diversity was evaluated based on sequences of ITS1 ribosomal DNA and COI mitochondrial DNA genes (Holguin et al., 2015). Although the evolutionary relationships of *Hoplolaimus* spp. have been studied, phylogenetic analyses of this genus within the subfamily Hoplolaiminae are still lacking. Therefore, this study aimed to characterize a population of a *Hoplolaimus* isolated in Namibia using both morphological and molecular approaches, which is presented herein as *H. pararobustus.* To our knowledge, this is the first report of *H. pararobustus* from maize (*Z. mays*) roots.

## Materials and methods

### Nematode extraction and morphological studies

Nematodes were extracted from roots using an adapted sugar centrifugal flotation method (Marais et al., 2017). Females and males were fixed in a heated 4% formaldehyde plus 1% propionic acid (FPG) solution, dehydrated in a glycerine solution, and mounted in glycerine on glass slides using a wax ring method (Marais et al., 2017). Measurements and drawings of the mounted specimens were done with a Nikon LABOPHOT-2 microscope equipped with a Nikon 1.25× drawing tube. All measurements were done at ×1,000 magnification. Curved structures were measured along the median line. Morphometrics were used in the descriptions with standard morphometric calculations and terms used throughout the paper (Siddiqi, 2000). Specimens were deposited in the National Collection of Nematodes (NCN), Biosystematics, Agricultural Research Council (ARC) – Plant Health and Protection (PHP), Pretoria.

### DNA extraction, PCR reaction, and gel electrophoresis

DNA from previously selected living males and females was extracted using chelex-100 as described by Rashidifard et al. (2019). Polymerase chain reaction (PCR) conditions followed the protocol of Swart et al. (2020) with the following DNA markers used for DNA amplification: 28 S rDNA: D2A (5–ACAAGTACCGTGAGGGAAAGTTG–3), D3B (5–TCGGAAGGAACCAGCTACTA–3) (Subbotin et al., 2006), and 18 S rDNA: SSU F04 (GCTTGTCTCAAAGATTAAGCC), SSU R26 (CATTCTTGGCAAATGCTTTCG) (Blaxter et al., 1998). DNA of the nematode specimens was stained using GelRed, loaded on 1% agarose gel, and visualized under UV transilluminator before sequencing by Inqaba Biotec (Pty) Ltd South Africa.

### Taxonomy and phylogenetic analyses

The newly generated 18 S and 28 S rDNA sequences of the Namibian nematode population were compared to those available in GenBank using a BLAST search. For phylogenetic tree construction, available sequences of the subfamily Hoplolaiminae were retrieved from GenBank for 18 S and 28 S data sets. Both data sets were aligned using MUSCLE (Edgar, 2004) in Geneious Prime 2020.0.4 (https//:www.geneious.com). The jModelTest 2.1.10 program (Darriba et al., 2012) was used to identify the best nucleotide substitution model. The General Time Reversible with an invariable site and a gamma distribution (GTR + I + G) and General Time Reversible with a Gamma distribution (GTR + G) were the most suitable models for 18 S and 28 S data sets, respectively. Bayesian analysis was accomplished using MrBayes 3.2.2 (Huelsenbeck and Ronquist, 2001) in Geneious Prime 2020.0.4 (https//:www.geneious.com); the chain was run for 3×10^6^ generations for each locus. After discarding burn-in samples (25%), posterior probability (PB) of the Bayesian trees was estimated using the Markov chain Monte Carlo (MCMC) algorithm (Larget and Simon, 1999) based on the 50% majority rule. *Heterodera schachtii* Schmidt, 1871 and *Globodera rostochiensis* (Wollenweber, 1923) Skarbilovich, 1959 were, respectively, used as outgroups for the 18 S and 28 S phylogenetic trees.

## Results

### Systematics

**Figure 1:**
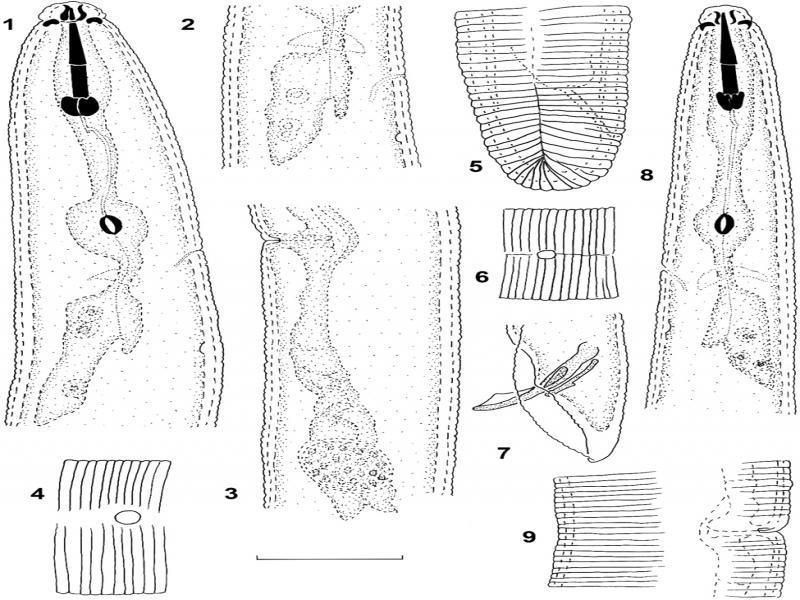
*Hoplolaimus pararobustus*. Female 1: Anterior part of the body; 2: Oesophageal overlap with an excretory pore; 3: Vulval area with posterior spermatheca; 4: Phasmid and lateral field in the anterior part of the body; 5: Tail with the lateral field; Male: 6: Phasmid with the lateral field at the posterior end; 7: Posterior end with spicules and bursa; 8: Anterior part of the body; 9: Lateral field opposite vulva. Scale bar = 20 µm.

*Hoplolaimus pararobustus* (Schuurmans Stekhoven and Teunissen, 1938) Sher, 1963 (Figure 1 and Table 1).

**Table 1.** Measurements of *Hoplolaimus pararobustus* females and males from maize in Namibia.

	*Hoplolaimus pararobustus* (Namibian specimens)		*Hoplolaimus pararobustus* (acc. to Fortuner (1991)		*Hoplolaimus pararobustus* (acc. to Van den Berg and Quénéhervé, 2012)		*Hoplolaimus columbus* (acc. to Fortuner, 1991)		*Hoplolaimus dubius* (acc. to Chaturvedi and Khera, 1979; Zarina and Maqbool, 1998)		*Hoplolaimus galeatus* (acc. to Van den Berg and Quénéhervé, 2012)		*Hoplolaimus seinhorsti* (acc. to Van den Berg and Quénéhervé, 2012	*Hoplolaimus seinhorsti* (acc. to Mansourabad et al., 2016)
Characters	Females	Males	Females	Males	Females	Males	Females	Males	Females	Males	Females	Males		
n	21	21	17	14			20	8				8		
L	1,100±76.1 (957–1,245)	925±65 (818–1,018)	1,314±0.127 (910–1,800)	1,158±0.102 (930–1,500)	940–1,800	920–1,500	1,260–1800	1,150–1,400	780–1,600	940–1,100	1,100–1,940	1,050–1,560	1,000–1,600	1,480–1,738
a	29±3.5 (23.7–36)	29.2±4.2 (22–38.3)	27.3±2.689 (20–39)	29.2±2.2227 (21–37.2)	20–39	21–37.2	30–38	31.9 (25.9–39.2)	31.94–51.29	32.78–43.87	22–34	23–32	24.1–40	25.5–33.8
b	7.4±0.7 (6.2–8.6)	6.7±0.4 (6–7.5)	8.98±1.361 (6–14)	8.5±1.328 (6.2–13.8)	6–14.1	6.2–13.8	9.1–12.4	10.9 (9.58–12.18)	8.16–12.49	8.0–9.45	6.3–10.8	8.3–10.3	5.2–14.2	
b’	9.5±0.8 (7.9–11)	8.5±0.7 (7.1–10.1)	7.1±0.682 (5.1–10)	6.6±0.57 (5–8.7)	–	–	6.3–9.7		6.04–9.42	5.95–6.76				
c	59.7±10.5 (46.9–80.6)	34.7±4.3 (25–41)	60.8±15.185 (10–164)	36.9±6.605 (22.2–51.9)	40–164	22.2–51.9	39–57	29.9 (26.8–33.1)	1.321.41	35.08–41.21	0.6–0.7	1.5–1.7	38–74	
c’	0.8±0.1 (0.6–1.1)	1.5±0.2 (1.3–2)	0.67±0.135 (0.4–0.9)	1.6±0.206 (1.4–2.1)	0.4–0.9	1.4–2.1				0.61–1.17			0.7–0.9	
o (%)	13±2 (8–17)	13±2.5 (8–18)	–	–	6.8–13.4	7.1–12.5	9–13	5.2 (4.8–5.2)	7.55–16.28	12.5–17.7	8.5–17	10–17		
M	51±1.5 (48–54)	51±1.8 (50–53)	–	–	–	–								
V (%)	55±2.5 (49–67)	–	52.68±2.609 (51–62)		51–62		51–60		41.56–59.16		52–60			
OV1 (%)	31±14.0 (18–51)	–												
OV2 (%)	–	–												
h	–	13±2.5 (10–19)												
Annulus width	2±0.3 (2–3)	2±0.3 (2–3)	2						2–3					
Lip region height	7±0.5 (6–8)	6±0.6 (5–7)												
Lip region width	13±1 (12–15)	12±0.9 (11–13)												
Stylet length	36±1.6 (34–40)	34±1.3 (32–38)	42.3±2.378 (37.5–49)	40.8±2.673 (35–46)	37.5–49.7	35–48	40–48	42 (40.2–43.7)	34.4–42.4		41.5–54	40–48	38–45	
Metenchium length	18±1.1 (17–21)	17±0.6 (16–18)												
Telenchium length	18±0.8 (17–19)	17±1 (15–20)												
Stylet knob height	6±0.5 (5–7)	5±0.6 (4–6)												
Stylet knob width	6±0.8 (5–9)	6±0.5 (5–7)							6.0–6.8					
Dorsal gland opening posterior to stylet knobs	5±0.7 (3–6)	4±0.9 (3–6)	5.6±1.377 (4–8)						3.2–5.6					
Median bulb length	17±1.3 (15–20)	16±1.5 (13–19)												
Median bulb width	15±1.3 (13–17)	12±1 (11–15)												
Medan bulb valve length	6±0.7 (4–6)	5±0.7 (4–7)												
Median bulb valve width	4±0.6 (3–5)	4±0.4 (3–4)												
Excretory pore from anterior end	108±11.9 (82–123)	93±13.6 (74–121)												
Esophagus length	148±7.6 (136–161)	139±10.4 (124–163)												
Esophagus overlap	31±7.2 (15–44)	30±5.7 (20–38)												
Body width at excretory pore	33±3.5 (27–40)	26±2.4 (23–30)												
Body width at midbody	39±4 (32–46)	32±2.9 (27–38)												
Body width at anus/cloaca	25±2.2 (21–29)	17±1.3 (15–20)												
Position of excretory pore/Esophagus length (%)	73±7.5 (61–85)	67±9.6 (61–85)												
Position of excretory pore/Body length (%)	10±1.3 (8–12)	10±(8–13)												
Anterior genital track length	241±69.2 (171–301)	–												
Testes length	–	350±34.6 (322–400) (*n*=4)												
Spicule length	–	38±2.3 (35–42)		46.9±3.318 (40–57)		40–57		46.8 (36.6–52.5)		368–43.36		40–52		
Gubernaculum length	–	17±2.3 (11–20)		21.9±2.4296 (15.4–31)		15.4–31				14.4–18.4		20–28		
Capitulum length	–	11 ±0.8 (10–13)												
Phasmid diameter	4±0.3 (3–4)	3±0.5 (3–4)		5.3 (4.1–6)										
Anterior phasmid as % from anterior end	36±6.9 (28–40) (n=3)	36±3.5 (28–43)			22–52	23.7–50.4	29–47	38 (35.4–42.2)	23.90–41.34	21.18–44.35	23–46	29–45	24–46	
Posterior phasmid as % from anterior end tail	79±4.1 (70–85)	81±4.7 (72–87)			58–89	68.9–85.4	79–90	82 (79.7–83.2)	82.20–96.44	76.69–85.99	72–88	75–89	68–90	
Tail length	19±3.1 (15–24)	27±3.5 (22–35)		7–15	16–28	26.1–35.1					17.5–36.2	32.3–44.2	22–28	
Number of tail annules									12–18					

All measurements are given in μm.

### Description

#### Female

Habitus slightly curved ventrad, C-shape, S-shape or curved into a complete spiral with head and tail end overlapping. Body length of (957– 1,245 μm). Cuticular annules distinct about 2 µm wide. Lip region broadly rounded and well set off from body usually with four distinct annules but sometimes with three on the one side and four on the other side. Basal annulus sometimes larger than others. Longitudinal striae on basal annulus faint. Labial framework well sclerotized. Stylet well-developed (34–40 μm) long with metenchium and telenchium almost equal in length. Stylet knobs tulip-shaped with two or more projections anteriorly. Median bulb round, muscular (15–20 μm long), 13–17 μm wide with a prominent centrally located valve, 4–6 μm long and 3–5 μm wide. Nerve ring encircling the isthmus. Excretory pore situated from opposite anterior part of median bulb to opposite the middle of the esophagus, 82–123 μm from anterior end, at 61–85% of esophagus length. Esophagus with three nuclei in each of the three esophageal glands extending dorsally over the intestine. Hemizonid two annules long and situated from opposite excretory pore, nine annules posterior to it. Hemizonion not seen. Esophagus glands overlap 15–44 μm long. Vulva a transverse slit with epiptygma folded into the vagina. Spermatheca small, oval or round, empty or filled with rounded sperm. Lateral field reduced; very faint line to just breaks in striae. Two or three very faint incomplete incisures can sometimes be seen in the lateral field area. Caudalid not seen. Intestine does not overlap the rectum. Phasmids: two enlarged scutella situated anterior and posterior to the vulva (3–4 μm in diameter). Tail short (15–24 μm), rounded with 6–12 annules.

#### Male

Habitus conforms to that of females, body 818–1018 μm long. Lip region rounded with three or four annuli, slightly offset from the body. Position and morphology of excretory pore, hemizonid, stylet, lateral field and phasmids similar to that of the females. Stylet slightly shorter, 32–38 µm long. Testis 322–400 μm long. Spicules ventrally arcuate (35–42 μm), gubernaculum protrusible through the cloaca with titillae (11–20 μm). Bursa large with crenate margin and enclosing the conoid tail tip.

### Diagnosis and relationships

*Hoplolaimus pararobustus* belongs to the group in which the lateral field is degenerate, not showing the regular compliment of four incisures and three and not six esophageal gland nuclei (Van den Berg and Quénéhervé, 2012). The current Namibian specimens are considered to be *H. pararobustus* because of the presence of a well set off, broadly round lip region with four annuli, lateral field represented by a single incisura, and two or three very faint incomplete incisures that can sometimes be seen in the lateral field area. Esophagus with three nuclei in three pharyngeal glands, no overlap of the rectum, and presence of males. The Namibian specimens correspond to the descriptions of Fortuner (1991), Larizza et al. (1998), and Van den Berg and Quénéhervé (2012), but for the lower range of stylet length of females (34–40 µm vs 37.5–49 µm) and males (32–42 µm vs 35–46 µm), expansion of the reported range for the position of the vulva (V = 49–67% vs V = 51–62.1%), and a shorter spicule (35–42 µm vs 40–57* *µm) than that reported (Van den Berg and Buckley, 1987; Fortuner, 1991; Larizza et al., 1998). Molecular analyses indicated that *H. columbus* Sher, 1963 is the closest to the Namibian population of *H. pararobustus. Hoplolaimus columbus* was described from soybean in the USA and is currently reported from India, Pakistan, Vietnam, Egypt, and the USA (Shafiee and Osman, 1971; Fortuner, 1991). This lance nematode belongs to the group in which the lateral field is represented by one indistinct incisure and six esophageal gland nuclei, one or two sometimes indistinct (Fortuner, 1991), this is in contrast with the three nuclei observed in the Namibian specimens. The current *H. pararobustus* female specimens differ from *H. columbus* females in short body length (957–1,245 µm vs 1,260–1,800 µm), shorter stylet length (34-40 µm vs 40-48 µm; b-value (6.2–8.6 vs 9.1–12.4)). The Namibian *H. pararobustus* differs from *H. columbus* males in shorter body length (808–1,018 µm vs 1150–1,400 µm), shorter stylet length (32–38 µm vs 40.2–43.7 µm), b-value (6–7.5 vs 9.58–12.18), and o-value (8–18 vs 4.8–5.2). The constructed Bayesian tree showed that *H. pararobustus* also grouped in a clade with *H. galeatus* that is representative of the group of species with four incisures in the lateral field and three esophageal gland nuclei (Van den Berg and Quénéhervé, 2012). *Hoplolaimus seinhorsti* reported from Africa, Asia, and Central and South America is representative of a group with one or no incisures in the lateral field and with six pharyngeal gland nuclei.

### Molecular characterization

**Figure 2:**
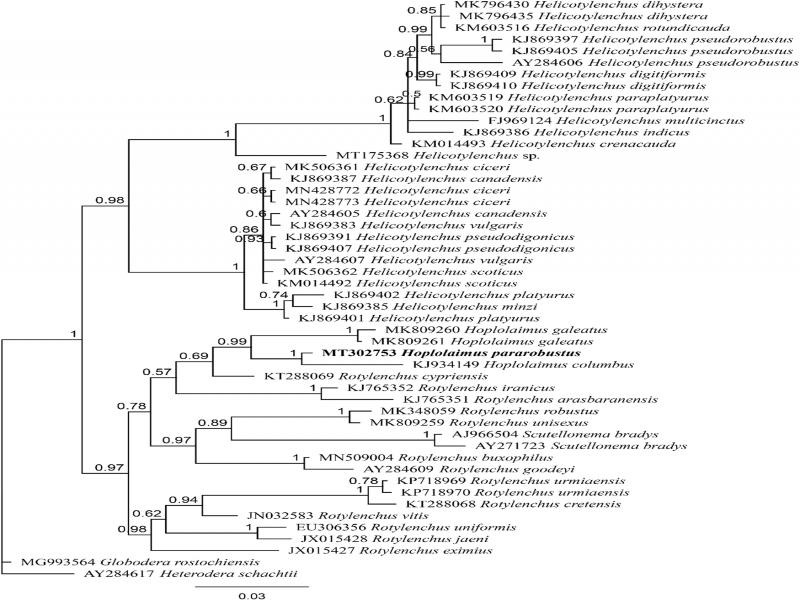
Bayesian tree inferred from partial 18 S rDNA sequence of *Hoplolaimus pararobustus* obtained from the rhizosphere of maize from Namibia under GTR + I + G (partition = 010020; lnL = 3717.3614; rAC = 1.0000; rAG = 2.0466; rAT = 1.0000; rCG = 1.0000; rCT = 5.6942; rGT = 1.0000; p-inv = 0.4370; gama shape = 0.3390) based on 50% majority role. The newly obtained sequence is indicated by bold font.

**Figure 3:**
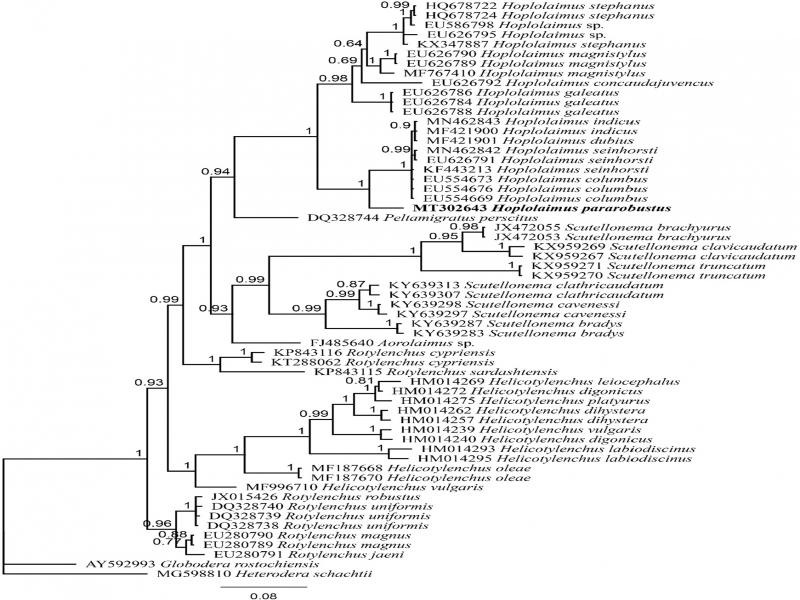
Bayesian tree inferred from partial 28 S rDNA sequence of *Hoplolaimus pararobustus* obtained from the rhizosphere of maize from Namibia under GTR + G (partition = 012314; lnL = 5311.5111; rAC = 0.9205; rAG = 4.5584; rAT = 2.2804; rCG = 0.4018; rCT = 4.5584; rGT = 1.0000; gamma shape = 0.2940) based on 50% majority role. The newly obtained sequence is indicated by bold font.

Nucleotide BLAST search using partial 18 S sequence of *H. pararobustus* (MT302753, 908 bp) showed the maximum identity of 98.4% to *Hoplolaimus* sp. (MK292131) and 98.1% to *H. galeatus* (KJ934131). BLAST search based on a partial 28 S sequence of *H. pararobustus* (MT302643, 700 bp) revealed a maximum identity of 96.6% to *Hoplolaimus* sp. (KY639326) and 95.9% to a population of *H. seinhorsti* Luc, 1958 (KF443213). During this study, one 908-bp-long 18 S sequence (MT302753) and one 700-bp-long 28 S sequence (MT302643) were obtained and deposited into GenBank. The 18 S alignment includes 50 sequences with 777 nucleotides in length, while the 28 S alignment includes 60 sequences with 644 nucleotides in length. The constructed Bayesian tree using 18 S data set showed that *H. pararobustus* is in a well-supported clade with two populations of *H. galeatus* and one population of *H. columbus* with *H. columbus* being the closest to the Namibian population of *H. pararobustus* (Figure 2). The inferred Bayesian tree using the 28 S data set indicated that *H. pararobustus* is in a maximally supported sister relation with *H. columbus, H. dubius* Chaturvedi, Singh & Khera, 1979, *H. indicus* Sher, 1963, and *H. sienhorsti* of which *H. columbus* was the closest taxa to the Namibian population of *H. pararobustus.* The molecular phylogeny of the Hoplolaiminae subfamily also indicated that, based on 18 S and 28 S data sets, *Rotylenchus* Filipjev, 1936 and *Peltamigratus* Sher, 1963 were the closest genera to the genus *Hoplolaimus*, respectively (Figure 3).

## Conclusion

A population of *H. pararobustus* was recovered from maize roots in Namibia. *Hoplolaimus pararobustus* belongs to the group in which the lateral field is degenerate, not showing the regular complement of four incisures and with three and not six esophageal gland nuclei (Van den Berg and Quénéhervé, 2012). The Namibian specimens correspond to the redescription of Fortuner (1991), but the lower range of the body and stylet length of females and males, the position of the vulva more anterior, and spicule length is shorter than that previously reported (Fortuner, 1991; Larizza et al., 1998; Van den Berg and Quénéhervé). *Hoplolaimus pararobustus* is commonly found in Namibia, reported from both cultivated and noncultivated areas, but this is according to our knowledge the first report from maize roots. This study reported the first molecular characterization of an African population of *Hoplolaimus*, in this case *H. pararobustus*. We resolved the evolutionary relationship of *H. pararobustus* based on partial 18 S and 28 S rDNA sequences. The constructed Bayesian tree using 18 S data set showed that *H. pararobustus* is in a well-supported clade with two populations of *H. galeatus* and one population of *H. columbus*, with *H. columbus* being the closest to the Namibian population of *H. pararobustus*. Ultimately, the monophyletic nature of the genus was confirmed using both phylogenetic trees.
